# Preface: special topic on topological matter

**DOI:** 10.1093/nsr/nwz032

**Published:** 2019-03-19

**Authors:** Fu-Chun Zhang

**Affiliations:** 1 Professor, Kavli Institute for Theoretical Sciences, University of Chinese Academy of Sciences, China; 2 Guest Editor of Special Topic & Editorial Board Member of NSR

Topological matter has now become a very broad field in frontier of condensed-matter physics and beyond. Initial study of quantum matter started about four decades ago when the quantum Hall effect was discovered. A quantum Hall state is characterized by a Chern number, which leads to the observed quantized transverse conductance in a 2D electron system under perpendicular high magnetic fields. Explosive development of the present topic of quantum matter was largely due to the theoretical work of Charles Kane and Gene Mele, and Shoucheng Zhang and their collaborators, who proposed topological insulators with time-reversal symmetry and predicted materials. The concept of topological matter with time-reversal symmetry may be traced back to a toy model studied earlier by Duncan Haldane. In the past 12 years, we have witnessed remarkable development in the field, thanks to the theoretical predictions and experimental discoveries in various types of topological matter.

In a personal interview on this special topic, Xiao-Gang Wen at MIT—a pioneer and chief architect of a new concept of ‘topological order’—examines its significance. Topological order describes quantum entanglement well beyond Landau's phase transition. Wen emphasizes the difference and close relation between the topological insulator and the topological order, and introduces the notion of symmetry-protected topology. In Wen's view, topological order may also describe the internal structure of empty space, hence making the world out of it. According to Jain's composite, fermion theory—a quantum-entangled fractional quantum Hall state of electrons—can be viewed as an integer quantum Hall effect of composite fermions without long-range entanglement. Therefore, I would expect a deep connection between the quantum entanglement and composite fermions.

The article by Ning Hao and Jiangping Hu is the first comprehensive review on the concept, models, materials and experiments on the topological states of iron-based superconductors. Topological states in iron-based superconductors are of great interest for the realization of Majorana modes at relatively high temperatures. This special topic includes several perspective articles. One of the important developments in topological matter is the observations of strong evidence of a Majorana zero mode inside corresponding superconducting vortices. Lingyuan Kong and Hong Ding report Majorana modes in iron-based superconductors. Yi Zhou examines theoretically spin-selective Andreev reflection of the Majorana zero modes, which was observed by the Jinfeng Jia group in 2016. In another perspective paper, Jian Wang discusses the observed tip-induced superconductivity in topological semimetal. Ke He and Qi-Kun Xue, who discovered the quantum anomalous Hall effect together with their collaborators in 2013, show an interesting perspective of quantum anomalous Hall hetero-structures. Chen Fang finds an automatic search for topological materials and his team have predicted non-trivial band topology in over 8000 materials, hence a large class of topological materials has been cataloged. Joel Moore reviews the optical properties of Weyl semimetals including TaAs and related compounds. Haizhou Lu proposes a 3D quantum Hall effect in topological semimetal and discusses the experimental findings and remaining questions on this. Yan Zhou outlines the intriguing physics and new spintronic device concept inmagnetic skyrmions.

This special topic only covers a small portion of the recent development in topological matter—a field expanding rapidly. I expect the field to continue to grow for years to come. The new challenges will be in understanding and predicting topological matter with strong interactions and in topological materials with great applications. China has been very active, with a number of major contributions in the field, including the discovery of the quantum anomalous Hall effect, the observations of Majorana zero modes inside superconducting topological vortices, and the theoretical predictions and experimental realization of topological materials such as the 3D topological insulators and Weyl semimetals. I fully expect continuous activities in this field from China and the world. Finally, I wish to thank all the authors, reviewers and editorial staff for their substantial support in the preparation of this special topic.

